# Crosstalk between Calcium and ROS in Pathophysiological Conditions

**DOI:** 10.1155/2019/9324018

**Published:** 2019-04-24

**Authors:** Simona Feno, Gaia Butera, Denis Vecellio Reane, Rosario Rizzuto, Anna Raffaello

**Affiliations:** Department of Biomedical Sciences, University of Padova, via U. Bassi 58/b, 35131 Padova, Italy

## Abstract

Calcium ions are highly versatile intracellular signals that regulate many cellular processes. The key to achieving this pleiotropic role is the spatiotemporal control of calcium concentration evoked by an extensive molecular repertoire of signalling components. Among these, reactive oxygen species (ROS) signalling, together with calcium signalling, plays a crucial role in controlling several physiopathological events. Although initially considered detrimental by-products of aerobic metabolism, it is now widely accepted that ROS, in subtoxic levels, act as signalling molecules. However, dysfunctions in the mechanisms controlling the physiological ROS concentration affect cellular homeostasis, leading to the pathogenesis of various disorders.

## 1. Calcium Homeostasis

Calcium ions (Ca^2+^) are one of the most crucial intracellular second messengers, involved in a plethora of cellular functions including cell survival and death, muscle contraction, regulation of metabolism, and gene expression [1]. To control these highly specialized functions, cells have developed sophisticated mechanisms to decode frequency-encoded Ca^2+^ signals [1].

The spatiotemporal regulation of cytosolic Ca^2+^ concentration ([Ca^2+^]_cyt_) relies on two key requirements. The first is the cooperation of two different sources of Ca^2+^ in the generation of [Ca^2+^]_cyt_ fluctuations: the extracellular medium, a virtually unlimited reservoir with a [Ca^2+^] of ∼1 mM [2], and the intracellular stores which are endowed with a [Ca^2+^] > 100 *μ*M, which allow rapid release of Ca^2+^ through store-resident channels [2]. The second requirement is the existence of a broad range of molecules that generate and decode [Ca^2+^]_cyt_ variations, such as pumps, channels, Ca^2+^-binding signalling molecules, enzymes, and buffering proteins [2].

Once having entered the cytosol, Ca^2+^ exerts its allosteric regulatory effects on many enzymes and proteins, impacting nearly every aspect of cellular life [3]. This is corroborated by the amount of energy that cells invest to maintain this strictly regulated [Ca^2+^]. Importantly, while complex molecules can be chemically altered, the only mechanism that exerts control over Ca^2+^ are chelation, subcellular compartmentalization and cell extrusion. The consequence is a very steep [Ca^2+^] gradient across the plasma membrane and the intracellular stores [3]. In resting cells, [Ca^2+^]_cyt_ are maintained within very low values of ∼100 nM, while the extracellular space generally presents a [Ca^2+^] of over 1 mM [2]. Different channels in the plasma membrane regulate Ca^2+^ entry from the extracellular space. Among these are the voltage-operated calcium channels (VOCCs), the receptor-operated calcium channels (ROCCs), the store-operated calcium channels (SOCCs), and the second messenger-operated calcium channels (SMOCs) that, according to the stimuli evoking channel activation, allow Ca^2+^ entry through the plasma membrane [3].

As mentioned above, Ca^2+^ is also efficiently stored in intracellular compartments that serve as the main sources of releasable Ca^2+^ for eliciting crucial cellular functions [3]. The most important intracellular store is the endoplasmic reticulum (ER) and its specialized counterpart in muscle cells, the sarcoplasmic reticulum (SR). In these compartments, [Ca^2+^] can reach ∼0.8 mM, depending on the cell type. Rapid release of Ca^2+^ from these compartments ensures [Ca^2+^]_cyt_ rises required for specific cellular functions [3] and is controlled by two large families of channels: the inositol 1,4,5-trisphosphate receptor (InsP3R) and ryanodine receptor (RYR) families [4].

The agonist of IP3R is generated by the phospholipase C (PLC) enzymatic activity. This enzyme usually undergoes a receptor-promoted activation, and it hydrolyses its substrate phosphatidylinositol 4,5-bisphosphate (PIP2) in diacylglycerol (DAG) and inositol 1,4,5-trisphosphate (InsP3) [5]. The interaction of InsP3 with its receptors (InsP3Rs) induces Ca^2+^ release to the cytosol [6]. Ca^2+^ itself regulates the InsP3Rs' open probability, activating InsP3Rs at increasing [Ca^2+^] up to a specific [Ca^2+^] threshold, above which further increases in [Ca^2+^] play an inhibitory function [2]. The InsP3R family displays a broad tissue distribution and comprises three isoforms, InsP3R1, InsP3R2, and InsP3R3, which show different expression profiles among different tissues. Of note, InsP3R1 is most abundant in the central nervous system (CNS) and InsP3R2 is ubiquitously expressed among tissues and is the most abundant isoform in cardiac muscle [7]. InsP3Rs form heterotetramers, whose activity displays unique properties and responsiveness to ATP, Ca^2+^, and InsP3 [8].

RyRs are structurally and functionally analogous to InsP3Rs, although they have approximately twice the conductance and molecular mass of InsP3Rs. RyRs are transmembrane proteins located in the ER/SR membrane, activated by the alkaloid ryanodine and by Ca^2+^ itself. Although Ca^2+^ is a major triggering ligand, several other players modulate RyRs' activity, such as the dihydropyridine receptor (DHPR; also known as L-type Ca^2+^ channel, Ca_V_1.1/1.2), protein kinase A (PKA), calmodulin (CaM), Ca^2+^/calmodulin-dependent protein kinase II (CaMKII), calsequestrin (CSQ), and the FK506-binding protein (FKBP12) [9]. Similarly to InsP3Rs, RyRs include three isoforms (RyR1-3), but, unlike the InsP3Rs that are widely expressed among tissues, RyR1-3 are almost exclusively expressed in excitable cell types. In detail, RyR1 is particularly enriched in skeletal muscle, RyR2 in cardiac muscle, and RyR3 is expressed more widely, although higher levels are found in the brain [6].

Once Ca^2+^ has carried out its signalling functions, it has to be rapidly removed from the cytosol by extrusion to the extracellular space or by compartmentalization to intracellular stores. This is achieved thanks to the activity of various pumps and exchangers, allowing intracellular [Ca^2+^] to return to its resting condition [3]. ATPase pumps compartmentalize Ca^2+^ into the ER/SR stores via the activity of ER/SR Ca^2+^ ATPase pumps (SERCAs) or extrude Ca^2+^ in the extracellular milieu via plasma membrane Ca^2+^ ATPases (PMCA pumps) by exploiting ATP-derived energy. A second mechanism utilizes the electrochemical gradient of Na^+^ across the plasma membrane to provide the energy to transport Ca^2+^ to the extracellular space through the Na^+^/Ca^2+^ (NCX) and Na^+^/Ca^2+^-K^+^ exchangers (NCKX) [3].

In addition, many studies have highlighted a role in regulation of [Ca^2+^]_cyt_ also for other membrane-bound compartments such as the Golgi apparatus, endolysosomes, and mitochondria [6]. Among these organelles, mitochondria are recognized as crucial regulators of cellular Ca^2+^ homeostasis. Indeed, mitochondrial Ca^2+^ uptake regulates many cellular processes, controlling the delicate balance between cell survival and death [2]. Moreover, mitochondrial Ca^2+^ buffering is involved in the control of Ca^2+^ gradient in defined cellular domains [2]. This is possible thanks to a strategic localization of mitochondria to the Ca^2+^ release units of the ER/SR that contributes to shape both the amplitude and the spatiotemporal patterns of cellular Ca^2+^ responses [3].

### 1.1. Mitochondrial Ca^2+^ Signalling

Over the past 60 years, intense research has defined the basic properties of mitochondria in Ca^2+^ handling. These studies have highlighted the role of mitochondria in decoding the cytosolic Ca^2+^ oscillations and in the regulation of cellular Ca^2+^ homeostasis [3]. The first evidence that mitochondria can take up Ca^2+^ dates back to the 60s, when pioneering studies demonstrated that energized mitochondria can rapidly and efficiently accumulate Ca^2+^ [10, 11]. The formulation of the chemiosmotic theory, together with the measurement of the mitochondrial membrane potential (ΔΨ_m_), led to the concept of an energetically favourable Ca^2+^ uptake mechanism [12, 13]. The generation of an internal negative electrochemical gradient by the mitochondrial respiratory chain, indeed, provides the thermodynamic basis for cation accumulation into the organelle matrix [13]. However, further characterizations of the mitochondrial Ca^2+^ uptake demonstrated that, despite the high selectivity of the mitochondrial Ca^2+^ uniporter (MCU) for Ca^2+^, measured by direct mitoplast patch-clamp of mitoplasts (dissociation constant (Kd) ≤ 2 nM) [14], the apparent mitochondrial affinity for Ca^2+^ was very low at physiological [Ca^2+^] [15]. Since cytosolic [Ca^2+^] is about 10-100 nM in resting conditions and reaches values of 2-3 *μ*M during cell stimulation, the role of mitochondria in Ca^2+^ homeostasis was considered marginal. Therefore, the plasma membrane and the ER became the major players in the Ca^2+^ signalling scene [15]. The situation reversed when tools to perform reliable measurement of [Ca^2+^] in intact living cells were developed, allowing to uncover the role of mitochondria in Ca^2+^ handling [16, 17]. Indeed, while [Ca^2+^]_mit_ in basal resting condition is very low, comparable to the cytosolic one (10-100 nM), upon cell stimulation, mitochondria are able to rapidly and efficiently accumulate Ca^2+^ at levels that exceed that of the bulk cytosol that, in some cell lines, can reach also [Ca^2+^] of 100 *μ*M [16]. The discrepancy between the low affinity of mitochondrial Ca^2+^ uptake and the prompt response of mitochondria to [Ca^2+^] increases was later solved by the demonstration that mitochondria are located in close proximity to the Ca^2+^ channels that elicit the rise in [Ca^2+^]_cyt_, the InsP3Rs, and the RYRs on the ER and SR [18, 19]. Indeed, these quasi-synaptic junctions with the ER/SR membranes allow mitochondria to directly sense local high [Ca^2+^] compatible with the low affinity of the MCU and that dissipates rapidly, thus preventing mitochondrial Ca^2+^ overload or vicious Ca^2+^ cycling across the mitochondrial membrane [18, 19].

Although the process of mitochondrial calcium uptake is prevalently studied at the level of the solutes impermeable inner mitochondrial membrane (IMM), the ability of Ca^2+^ to cross the outer mitochondrial membrane (OMM) plays a crucial role. The OMM permeability to solutes is prevalently due to the high expression of the voltage-dependent ion channels (VDACs), permeable to solutes smaller than 5 kDa and, thus, also Ca^2+^ [20]. Three different VDAC isoforms, VDAC1, VDAC2, and VDAC3, have been identified. VDAC1, the best characterized isoform [21], acts as a mitochondrial gatekeeper, controlling the metabolic and energy crosstalk between the mitochondria and the rest of the cell [21]. Furthermore, it has been also shown that VDACs' expression levels can limit calcium accumulation inside the matrix. It was demonstrated, indeed, that VDAC overexpression augments agonist-dependent rises in [Ca^2+^]_mit_, whereas VDAC downregulation has the opposite effect [22, 23].

### 1.2. The Mitochondrial Ca^2+^ Uniporter Complex: Structural and Functional Complexity

The molecular identity of the protein responsible for mitochondrial Ca^2+^ uptake, MCU, was uncovered only in 2011 by two different groups [24, 25], marking a turning point in the study of the pathophysiological roles of mitochondrial Ca^2+^ uptake. The characterization of the MCU revealed that this channel is a high-molecular-weight complex composed of both pore-forming and regulatory subunits [1].

**Figure 1:**
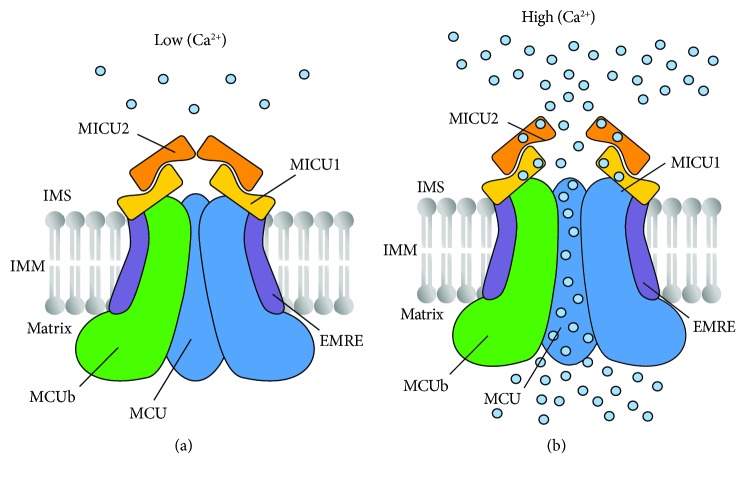
Schematic representation of the mitochondrial Ca^2+^ uniporter (MCU) complex. The MCU complex is composed by pore-forming subunits (that comprise the channel subunits MCU, the dominant-negative subunits MCUb, and the transmembrane regulator EMRE) and regulatory subunits (MICU1 and MICU2). MICU1 and MICU2 sense, through EF-hand domains, the increase of Ca^2+^ levels in the intermembrane space (IMS). In resting conditions (on the left), MICU1-MICU2 heterodimers act as gatekeeper of the channel, thus preventing Ca^2+^ vicious cycling and mitochondrial matrix overload. Increases in calcium concentration as a result of cell stimulation (on the right) not only release the inhibitory function of the MICU1-MICU2 heterodimers but also further stimulate MCU channel opening, ensuring the prompt response of mitochondrial metabolism to cell stimulation.

From the primary amino acidic sequence analysis, MCU consists of two transmembrane domains spanning the IMM (Figure 1 and [1]). Soon after its discovery, it was clear that the MCU was part of a macromolecular complex since it lacks classical Ca^2+^-binding domains and the loop region that faces the intermembrane space (IMS) appears to be too small to contain regulatory elements [1]. This was confirmed by blue native gel separation experiments of purified mitochondria that display a high-molecular-weight complex containing MCU with an apparent molecular weight of about 450 kDa, suggesting that many other proteins are part of the channel [24, 26–28].

Recently, the MCU protein structure was solved by different laboratories. First, MCU was shown to be a pentamer of the MCU homolog from *Caenorhabditis elegans* deleted of the N-terminal domain, which was defined by using nuclear magnetic resonance (NMR) and negative-stain electron microscopy [29]. Recently, four independent groups characterized the structure full-length *Fungi* homologs of MCU by Cryo-EM and/or X-ray diffraction approaches [30–33]. Unlike the previous study, they found a tetrameric architecture. Since these *Fungi* MCU homologs share only about 40% of similarity with metazoan MCU, prevalently conserved in the transmembrane regions and in the coiled-coil domains, Baradaran and coworkers performed Cryo-EM studies also on zebrafish MCU homolog, which displays a higher similarity with human MCU (91%). Although the resolution obtained is lower (8.5 Å), the overall structure is similar to that of *Fungi* MCU and also displays a tetrameric architecture [30]. Interestingly, the conserved DIME motif that connects the two transmembrane domains appears to be part of the second transmembrane domain and seems to confer Ca^2+^ selectivity to the MCU. The N-terminal domain is poorly conserved in these MCU homologs, but the human NTD of MCU was previously crystallized [34].

After the discovery of MCU, we have witnessed an explosion of studies aimed at clarifying the composition of the channel and the regulation of its activity. These studies demonstrated that three proteins compose the protein structure that spans the IMM: MCU, MCUb, and EMRE. Furthermore, three regulatory subunits were identified (MICU1, MICU2, and MICU3).

EMRE (“essential MCU regulator”) is a 10 kDa, metazoan-specific protein with a single transmembrane domain that spans the IMM with a highly acidic carboxyl terminus (Figure 1 and [28]). This protein has been proposed to play a dual function in the regulation of MCU activity. First, it seems required for MCU channel activity since its silencing abrogates mitochondrial Ca^2+^ uptake [28], although experiments in the planar lipid bilayer demonstrated that mouse MCU alone is sufficient to give rise to Ca^2+^ currents [25]. Second, EMRE seems fundamental in mediating the interaction between MCU and the regulatory subunits MICU1 and MICU2 [28], although it has also been observed that MICU1 is sufficient to induce MCU channel activity [26]. In addition, in yeast cells that do not present mitochondrial Ca^2+^ uptake, the *Dictyostelium discoideum* MCU homolog conducts Ca^2+^ in the absence of an EMRE homolog while human MCU requires the presence of EMRE to act as a functional channel [35]. Very recently, it was shown that the acidic C-terminal domain functions as a matrix Ca^2+^ sensor that regulates the MCU activity. In this model, EMRE acts, together with MICU1, as a regulatory complex able to sense [Ca^2+^] at both sides of IMM [36]. Nevertheless, these data were questioned by a study showing that EMRE displays a different topology across the IMM [37]. Future experiments will clarify the role of EMRE in the regulation of MCU channel activity.

MCUb is a MCU isoform conserved in most vertebrates and in many plants but absent in other organisms where the MCU is present (Figure 1 and [38]). MCU and MCUb share 50% sequence similarity, and each possesses two transmembrane domains separated by a short loop almost identical between the two [38]. Despite the huge sequence similarity in the transmembrane domains, MCUb displays altered ion permeation, given to two conserved amino acid substitutions in close proximity of the conserved DIME motif that drastically reduces the conductivity of the channel [38]. Specifically, the Arg 251 and Glu 256 residues are mutated in Trp and Val, respectively (R251W and E256V). These substitutions drastically reduce conductivity of the channel reducing [Ca^2+^]_mit_uptake [38]. According to this evidence, in living cells, the overexpression of MCUb reduces the amplitude of [Ca^2+^]_mit_ transients evoked by agonist stimulation and MCUb silencing elicits the opposite effect, suggesting that this protein acts as a dominant-negative subunit that incorporates into the channel and reduces its activity [38]. Interestingly, MCU and MCUb expression profiles widely differ among tissues, possibly providing an intrinsic regulatory mechanism to set the mitochondrial responsiveness to Ca^2+^-mediated signals in a defined cell type [38]. Consistently, tissues characterized by low mitochondrial Ca^2+^ transients, such as the heart, exhibit a low MCU/MCUb ratio, while others, such as skeletal muscle, display a higher ratio and high mitochondrial Ca^2+^ uptake levels [38].

### 1.3. MCU-Associated Regulators

One of the key features of mitochondrial Ca^2+^ uptake is its sigmoidal response to extra-mitochondrial [Ca^2+^]. At resting [Ca^2+^]_cyt_, mitochondrial Ca^2+^ uptake is inhibited, despite the steep ΔΨ_m_ [3]. This property prevents matrix Ca^2+^ overload and the dissipation of the ΔΨ_m_, leading to deleterious effects of Ca^2+^ cycling and matrix overload. At higher [Ca^2+^]_cyt_, when cells are stimulated, mitochondria have to respond promptly, increasing the Ca^2+^-carrying capacity [3]. The lack of Ca^2+^-sensing domains in the MCU protein sequence, as mentioned above, suggested the existence of a highly sophisticated gatekeeping mechanism, including both negative modulators, acting at low [Ca^2+^], and activators able to induce Ca^2+^ uptake during cell stimulation. Accordingly, it was shown that the regulation of the MCU complex activity is possible thanks to the MICU (mitochondrial calcium uptake) family of intermembrane space (IMS) proteins, composed by MICU1, MICU2, and MICU3 [39]. These three regulators share common features: they are localized to mitochondria, they display EF-hand Ca^2+^-binding domains in their protein sequence, and they interact with MCU (Figure 1) [39].

MICU1 (mitochondrial calcium uptake 1) was identified even before the identification of MCU as a critical modulator of mitochondrial Ca^2+^ uptake (Figure 1 and [40]). MICU1 was initially proposed to be required for mitochondrial Ca^2+^ uptake, since its silencing was sufficient to abolish mitochondrial Ca^2+^ entry in intact and permeabilized cells [40]. This evidence was questioned by other laboratories that showed that MICU1 silencing is sufficient to induce mitochondrial Ca^2+^ overload, suggesting that MICU1 could play a gatekeeping role in preventing mitochondrial Ca^2+^ uptake at low [Ca^2+^]_cyt_, while playing a minor role at higher [Ca^2+^]_cyt_ [41]. The identification of MICU1 loss-of-function mutations in patients affected by a disease characterized by proximal myopathy, learning difficulties, a progressive extrapyramidal movement disorder, and increased mitochondrial Ca^2+^ load supported this hypothesis [42]. The gatekeeper role of MICU1 was confirmed by Csordás and coworkers [43]. They also showed that silencing of MICU1 highly affects the cooperativity of mitochondrial Ca^2+^ uptake, thus hypothesizing that MICU1 could play a dual function depending on [Ca^2+^]_cyt_.

Very recently, Csordás et al.s' group dissected the mechanism that allows MICU1 to interact with MCU, to regulate mitochondrial Ca^2+^ entry and the sensitivity to ruthenium red/Ru360 (RuRed/Ru360), a compound that inhibits the activity of the uniporter [44]. Indeed, a structural and functional interaction of the DIME motif of MCU was reported, identified as the selectivity filter, with a domain of MICU1, named DID, as the DIME-interacting domain. The interaction between these two domains appears to be fundamental for ensuring both the threshold and cooperative activation of the MCU complex-mediated Ca^2+^ uptake and thus to avoid mitochondrial Ca^2+^ overload [44]. Furthermore, the DID motif limits the access of RuRed/Ru360 to its target site in the DIME domain of MCU since MICU1 removal can sensitize mitochondria to inhibition by this compound, thus predicting a different RuRed/Ru360 sensitivity of the MCU complex in various tissues, in light of recent data on tissue-specific differences in MICU1 abundance relative to MCU [45].

In addition to MICU1, other MCU complex components have also been discovered. Two paralogs of MICU1, originating from a gene duplication event prior to vertebrate evolution, were identified: MICU2 (Figure 1 and [27]), which displays a tissue expression pattern similar to that of MICU1, and MICU3, whose expression is restricted to the nervous system (NS) and, at lower levels, to the skeletal muscle [27].

MICU2 discovery [27] helped to clarify the mechanism responsible for the sigmoidal response of the MCU to extramitochondrial [Ca^2+^] that allows on the one hand minimal Ca^2+^ uptake in the presence of a very large driving force for cation accumulation thus preventing mitochondrial Ca^2+^ overload and on the other hand ensures rapid Ca^2+^ accumulation during cell stimulation. Importantly, MICU2 protein stability depends on that of MICU1 [26, 27, 46], since MICU1 silencing induces MICU2 protein degradation, suggesting that the effect of MICU1 silencing on mitochondrial Ca^2+^ uptake could be due also to the concomitant disappearance of MICU2 protein. Notably, MICU1 and MICU2 have been shown to form an obligate heterodimer through the formation of a disulphide bond [26], which is regulated by the mitochondrial oxidoreductase Mia40 [47].

MICU2 was demonstrated to act as the genuine gatekeeper of the MCU at low [Ca^2+^]_cyt_ [26]. As soon as extramitochondrial [Ca^2+^] increases, Ca^2+^-dependent MICU2 inhibition and MICU1 activation guarantee the prompt response of rapid mitochondrial Ca^2+^ accumulation (Figure 1 and [26]).

Recently, an alternative splice variant of MICU1, named MICU1.1, was identified and characterized [48]. It has been shown that the expression of this splice variant varies greatly among tissues. Indeed, MICU1.1 is present only in skeletal muscle, where it is the predominant isoform, and lower levels are found in the brain, suggesting tissue-specific functions. MICU1.1 is characterized by the addition of a micro-exon coding for four amino acids (EFWQ) far from the EF-hand domains, which greatly modifies the properties of the protein. In detail, MICU1.1 can bind Ca^2+^ one order of magnitude more efficiently than MICU1 and, when heterodimerized with MICU2, activates MCU current at lower [Ca^2+^] than MICU1-MICU2 heterodimers [48].

How the MICU1.1 extra exon impact on MICU1 structure and modifies the Ca^2+^-binding affinity of the EF-hand domains remains unaccounted. In this regard, the domain that contains the extra exon was not resolved in the MICU1 crystal structure, suggesting that it is part of a highly flexible region [49]. This can suggest a putative role of this protein domain in protein-protein interactions, which can modify the MICU1 modulatory properties.

It was hypothesized that the inclusion of this splice variant in the MCU complex could represent an important mechanism in excitable tissues, where fast Ca^2+^ transients occur. Indeed, in skeletal muscle, the prevalent expression of MICU1.1 allows a prompter response of mitochondria metabolism to [Ca^2+^] [48], ensuring a sustained ATP production during contraction, since mitochondrial Ca^2+^ positively regulates the activity of three key dehydrogenases of the tricarboxylic acid (TCA) cycle: pyruvate, isocitrate, and *α*-ketoglutarate dehydrogenases [50].

MICU3 shares a mitochondrial targeting sequence (MTS) at the amino terminus and two canonical Ca^2+^-binding EF-hand domains with MICU1 and MICU2 [27]. MICU3, unlike MICU1 and MICU2 that present a ubiquitous and strongly correlated expression pattern among tissues, is expressed only in the CNS and, at low levels, in skeletal muscle [27]. Recently, it was shown that MICU3 exists in a disulfide bond-mediated dimer only with MICU1 but not with itself or MICU2 and acts as a highly potent stimulator of MCU activity, with no gatekeeping function [51]. In this regard, it was shown that neurons simultaneously express both MICU1-MICU2 and MICU1-MICU3 heterodimers. The first avoids low vicious Ca^2+^ cycling in resting conditions; the latter anticipates MCU opening, activating organelle Ca^2+^ uptake even in the presence of small and rapid cytosolic Ca^2+^ signals. Thus, MICU3 in neurons allows enhancing MCU opening in order to guarantee organelle Ca^2+^ uptake also in response to small and fast increases of [Ca^2+^]_cyt_ [51].

Finally, MCUR1, an IMM-integral protein, was initially reported to function as a regulator of the MCU complex [52], although this protein was not among the MCU interactors [28]. Furthermore, MCUR1 has a homolog in *Saccharomyces cerevisiae*, an organism that lacks mitochondrial Ca^2+^ uptake. Its role in MCU complex regulation is highly debated, since it has been shown, both in yeast and mammalian cells, that it is involved in complex IV assembly [53]. Furthermore, it has been reported that MCUR1 silencing causes a consistent drop of ΔΨ_m_, with consequent reduction of mitochondrial Ca^2+^ uptake [53].

Moreover, recently, Chaudhuri and coworkers found discordant results. Indeed, they found no significant changes in ΔΨ_m_ and no changes in mitochondrial Ca^2+^ uptake rates after manipulating MCUR1 expression, but they demonstrated that MCUR1 regulates the amount of Ca^2+^ required to induce the permeability transition [54].

Therefore, whether this protein controls directly the activity of MCU or whether it affects mitochondrial Ca^2+^ uptake by indirectly impinging on mitochondrial bioenergetics is still highly debated.

## 2. Ca^2+^ and ROS as a Mutual Interplay

The understanding of the role of mitochondria as integration points of different cellular signals, and the mechanisms through which mitochondria translate these stimuli in biological responses, represents a new challenge in biomedical research. As discussed above, the ability of mitochondria to accumulate Ca^2+^ is fundamental for tissue homeostasis [1]. However, mitochondrial Ca^2+^ overload leads to reduced ATP production and sustained opening of the mPTP, a high conductance channel, whose opening enables the release of proapoptotic mitochondrial components [55].

Matrix Ca^2+^, beyond a certain threshold, together with other causal factors, most notably oxidative stress, high phosphate concentrations, and low adenine nucleotide concentration, is an essential permissive factor for mPTP opening [55]. This event triggers the so-called mitochondrial permeability transition that is characterized by a dramatic increase in the mitochondrial membrane permeability to any molecule smaller than 1.5 kDa. The consequent dissipation of the mitochondrial ΔΨ_m_ leads to membrane depolarization and mitochondrial swelling, increased mitochondrial reactive oxygen species (mROS) generation, cytochrome c release, and apoptosis [55].

The molecular identity of the mPTP is still debated. It was proposed that the adenosine nucleotide translocase (ANT), VDAC, and the translocator protein (TSPO) are essential components of the mPTP [54]. However, biochemical characterization to knockout models of these proteins suggests that they are dispensable for mPTP activity [56]. It has been recently proposed that mPTP is generated at the interface of two adjacent monomers of the F-ATP synthase through a strictly Ca^2+^-dependent mechanism, since gel-excised dimers of F-ATP synthase rapidly give rise to mPTP-like channels in lipid bilayers [57, 58]. Nevertheless, the mechanism of PTP formation and activation is still debated. Detailed discussion of this aspect is beyond the scope of this review, and readers are referred to specific contributions on this topic [59–65].

### 2.1. Mitochondrial ROS Production and Regulation

Mitochondria, through the respiratory chain, especially complexes I and III, are considered the main source of physiological ROS [66]. mROS are generated in both physiological and pathological conditions [66]. Indeed, on the one hand moderate levels of ROS are involved in cell signalling by affecting the redox state of signalling proteins, but on the other hand, when in excess, mROS are among the major determinants of toxicity in cells and organisms [66].

**Figure 2:**
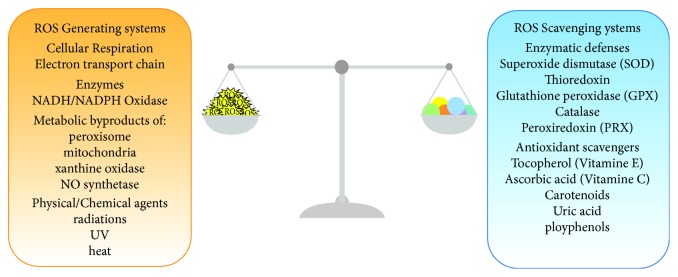
ROS production and scavenging systems. In physiological conditions, the balance between ROS generation and ROS scavenging is highly controlled. The energy production pathways (TCA cycle and OXPHOS), enzymatic reactions, by-products of metabolic pathways, and physical or chemical agents can lead to ROS production. As for the ROS-scavenging mechanisms, enzymatic defences and antioxidant scavengers neutralize the free radical reactions. When an imbalance between ROS production and ROS scavenging occurs, cells undergo oxidative stress, leading to severe cellular damage, cell death, and consequently whole organ and organism failure.

During respiration, superoxide (O_2_^−^) is produced by partial reduction of molecular oxygen. Subsequently, hydrogen peroxide (H_2_O_2_) is formed by the action of matrix antioxidant defence enzymes as superoxide dismutase (SOD) [66]. H_2_O_2_ is transformed in water by glutathione peroxidase (GPX), peroxiredoxin (PRX), and catalases [66]. The regulation of the activity and the expression levels of these antioxidant enzymes are controlled by a plethora of mechanisms [66]. Under physiological conditions, the balance between ROS generation and ROS scavenging is highly controlled (Figure 2). Physiological ROS levels initiate a wide array of cellular responses, ranging from triggering signalling pathways, activation of mitochondrial fission and autophagy, adaptation to hypoxic condition, and differentiation to regulation of aging-related processes [67]. In these specific conditions, ROS production is induced in response to a stress and it functions as an intermediate signalling to facilitate cellular adaptation [68].

ROS production, when not compensated by ROS scavenging, results in oxidative stress leading to severe cellular damage and cell death [67]. In this condition, ROS become causative of several pathological states by the direct modification of cellular macromolecules, leading to alterations of the redox state of factors involved in signal transduction, inducing either hyper- or hypofunctionality of several signalling pathways [67, 68]. Oxidative stress has been shown to be at the basis of aging and many pathological disorders. Indeed, ROS are responsible of cell death in pathological conditions such as myocardial infarct or stroke [67].

In physiological conditions and in a tissue-specific manner, mitochondrial Ca^2+^ uptake, by impinging on Krebs cycle enzymes and electron transport chain (ETC) activity, generates a ROS signals [69]. This signalling axis operates within a physiological window of [Ca^2+^]. Therefore, when [Ca^2+^] overcomes this threshold, mROS production becomes detrimental and compromises mitochondrial bioenergetics and cell functions [70, 71]. Mitochondrial Ca^2+^ may promote mROS formation both directly, by stimulating mROS-generating enzymes, like glycerol phosphate and *α*-ketoglutarate dehydrogenase, and indirectly, as in the case of nitric oxide synthase (NOS) activation that, by forming NO, blocks complex IV, leading to excessive mROS formation [70].

Finally, mitochondrial Ca^2+^ overload triggers mPTP opening. Indeed, [Ca^2+^]_cyt_ increases beyond a certain value and induces mitochondrial Ca^2+^ overload, triggering the mitochondrial “permeability transition.” In this condition, the mitochondrial membrane becomes permeable to any molecule less than 1.5 kDa in size. Consequent dissipation of ΔΨ_m_ leads to a permanent membrane depolarization, decreased ATP production, and eventually cell apoptosis. Moreover, mitochondrial membrane depolarization leads to crista unfolding, uncoupling of oxidative phosphorylation, and the reverse electron transport (RET). RET is evoked when electrons from ubiquinol are transferred back to respiratory complex I, reducing NAD^+^ to NADH. This process generates a significant amount of ROS [56].

Since mitochondrial Ca^2+^ plays a key role in ROS production, the cellular redox state can also significantly modulate Ca^2+^ signalling [70, 71]. Indeed, it has been clearly demonstrated that redox equilibrium controls a variety of receptors, proteins, and other signalling molecules that, in turn, might directly or indirectly modify components of Ca^2+^ signalling pathways, thus altering Ca^2+^ homeostasis and reshaping local and global Ca^2+^ signals [70]. When the redox equilibrium is disturbed, due to the excessive accumulation or clearance of ROS, many cellular signalling pathways are influenced, leading to cellular dysfunction and subsequently to the development of various pathologies, including neurodegenerative disorders, cancer, diabetes, atherosclerosis, and ischemia/reperfusion (I/R) injury. Therefore, both mROS and mitochondrial Ca^2+^ signalling are two functional entities that strictly cooperate in order to contribute to the maintenance of cellular homeostasis [71].

Intriguingly, Dong and coworkers analysed the crosstalk between intracellular ROS levels and [Ca^2+^]_mit_, suggesting that oxidative stress, and thus ROS accumulation, plays a positive feedback role in modulating MCU activity [72]. Indeed, they observed that MCU activity increases in cells exposed to endotoxin-mediated oxidative stress, leading to augmented [Ca^2+^]_mit_ at resting [Ca^2+^]_cyt_. In detail, they identified a conserved cysteine in metazoan at position 97 (Cys-97) in the NTD of the MCU protein sequence to be the only reactive thiol in human MCU that undergoes redox modification (S-glutathionylation). The Cys-97 residue is surface-exposed and primed for an oxidative posttranslational modification that induces a conformational change of MCU that promotes the clustering of MCU channels and their persistent activation [72]. These data suggest that, in condition of oxidative stress, mROS overproduction in the mitochondrial matrix perturbs mitochondrial antioxidant activity resulting in S-glutathionylation of MCU Cys-97. The conjugation of glutathione causes a conformation change within the N-terminal domain that appears to promote MCU channel activity in resting condition. The increased MCU activity, in turn, enhances the production of mROS in the mitochondrial matrix in a positive feedback mechanism, thus leading to perturbation of mitochondrial bioenergetics and cell functions [72]. Overall, these data strongly suggest that ROS and mitochondrial Ca^2+^ signals are intimately interconnected, leading to a specific and adaptive response to given stimuli.

**Figure 3:**
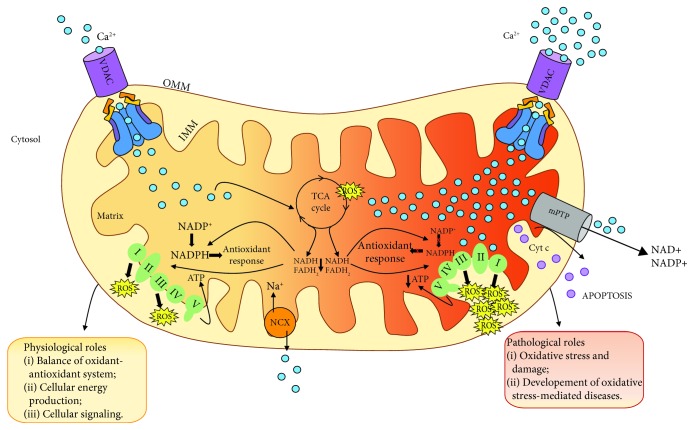
Regulation of mitochondrial Ca^2+^ uptake controls energy metabolism, mtROS production, and cell death. mtROS represent a by-product of oxidative phosphorylation and exert a beneficial or detrimental effect depending on their concentration and on the biological contest. In physiological conditions, mitochondrial Ca^2+^ uptake stimulates the TCA cycle and ATP production (left part). At physiological [Ca^2+^]_mit_, the amount of ROS is counteracted by the activity of the antioxidant system (left part). In pathological conditions, excessive Ca^2+^ accumulation by mitochondria (right part) increases mtROS production which, in turn, affects antioxidant response. Altogether, this leads to the increase in the mPTP open probability, leading to irreversible collapse of the mitochondrial membrane potential, swelling of mitochondria, and thus release of cytochrome c, culminating in cell death.

Excessive ROS are recognized as one of the causative factors in the development of a diverse array of diseases including cardiovascular, skeletal muscle, and neurodegenerative diseases and cancer progression (Figure 3 and [67, 71]). This review is aimed at describing some pathological conditions characterized by a dysregulation of mitochondrial Ca^2+^ uptake associated with an excessive ROS production.

## 3. Crosstalk of Mitochondrial Ca^2+^ Uptake and Mitochondrial Redox State in Physiopathology

### 3.1. Heart

Most of the ATP necessary for cardiac excitation and contraction is synthesized within mitochondria via oxidative phosphorylation which, as described above, is a process modulated by Ca^2+^ [1]. Furthermore, mitochondria are the major source of ROS that represent by-products of oxidative phosphorylation [73]. In living cells, and in particular in cardiac myocytes, ROS are also produced by extra-mitochondrial sources including NADPH oxidase, uncoupled NOS, xanthine oxidase, and monoamine oxidase [74]. In physiological conditions, ROS concentration is tightly regulated by antioxidants keeping them in a picomolar range. Low concentrations of ROS allow them to act as second messengers in signal transduction for vascular homeostasis and cell signalling [73]. In detail, the activity of redox-sensitive proteins, including Ca^2+^-handling proteins, contractile proteins, and proteins involved in various signalling pathways and in transcriptional activities, can be modulated by ROS [73].

Redox modulation of calcium-handling proteins directly affects cardiac contraction by altering intracellular calcium concentration [75]. In detail, ROS can oxidase and directly enhance the activity of Ca^2+^/calmodulin-dependent kinase II (CaMKII) that in turn phosphorylates and activates several Ca^2+^-handling proteins such as the cardiac ryanodine receptor RyR2 or cardiac SERCA [76]. Cardiac RyR2 mediates Ca^2+^ release to the cytosolic compartment from SR during excitation-contraction coupling (ECC) and is itself subject to oxidation that increases RyR2 open probability but may lead to irreversible activation and Ca^2+^ leak [77]. Similarly to RyR2, cardiac SERCA, which transfers Ca^2+^ from the cytosol to the SR at the expense of ATP hydrolysis during diastole, might be also directly regulated by oxidation. In particular, low oxidation levels reversibly increase SERCA activity whereas higher levels cause irreversible inactivation [77].

When the equilibrium between ROS production and scavenging is altered, ROS can cause damage to lipids, proteins, and DNA by contributing to the development and progression of cardiovascular diseases such as atherosclerosis, I/R injury, chronic ischemic heart disease, cardiomyopathy, heart failure, and arrhythmias [73]. As already discussed in the previous paragraph, excessive ROS levels is caused not only by defective ROS scavenging mechanisms but also by excessive ROS production.

Since cardiac mitochondria are the major producers of ROS through oxidative phosphorylation and Ca^2+^ plays a key role in promoting aerobic metabolism, dysregulation of mitochondrial Ca^2+^ homeostasis translates also in oxidative stress [73].

During myofibril contraction, ATP is hydrolysed to adenosine diphosphate (ADP) which moves into mitochondria through the adenine nucleotide transporter (ANT) and activates the F_1_F_0_-ATPase to regenerate ATP [78]. The increase in mitochondrial ADP content accelerates electron flux along the ETC and induces the oxidation of the reduced NADH and FADH_2_ which act as electron donors to sustain oxidative phosphorylation [78]. At the same time, Ca^2+^ is accumulated into the mitochondrial matrix through the MCU, where it stimulates the activity of the Krebs cycle to replenish the reduced pyridine nucleotides as NADH and FADH_2_ which act as electron donors and sustain oxidative phosphorylation [78]. Therefore, Ca^2+^ plays a dual role since it both increases electron flux along the ETC and regenerates energy by increasing the electron flow from the Krebs cycle to the ETC [79, 80].

The ability of mitochondria to cope the increase in energy demand, occurring during increased cardiac workload or hormonal stimulation, is due to the close apposition of mitochondria and the SR [3, 81, 82]. It was hypothesized that Ca^2+^ released from the SR will elevate local Ca^2+^ to high levels resulting in a large mitochondrial Ca^2+^ influx [81]. Nevertheless, direct patch clamp recordings demonstrated that cardiac mitochondria's MCU current (*I*_MCU_) is substantially smaller than that of other tissues, such as skeletal muscle, where *I*_MCU_ is 30 times bigger [83]. Notably, these two tissues present different mitochondrial volume fractions. Indeed, cardiac myocytes show one of the highest mitochondrial volume fractions in mammalian cells (37%), while in skeletal muscle this fraction is much lower (5%) [83]. Furthermore, also the MCU complex component stoichiometry is different between these two tissues. For example, in the heart, the expression of the dominant-negative subunit MCUb is higher than that in the skeletal muscle, inversely correlating with mitochondrial Ca^2+^ current [38]. In addition, mitochondrial Ca^2+^ uptake in the heart is controlled by a low MICU1/MCU ratio, thus lowering the threshold and cooperativity of the MCU complex [45]. It has been proposed that both the low Ca^2+^ conductivity of the MCU and the complexity of its regulatory components could represent protective mechanisms by which cardiac mitochondria regulate [Ca^2+^]_mit_ by preventing Ca^2+^ overload [83]. Indeed, an excessive mitochondrial Ca^2+^ uptake associated with an increase in ROS accumulation leads to the opening of the PTP, irreversible collapse of mitochondrial membranepotential, swelling of mitochondria, and thus release of cytochrome c resulting in necrotic cardiomyocyte cell death, a common scenario observed in the ischemic/reperfused myocardium [55].

In chronic heart failure (HF), perturbations of ECC cause contractile dysfunction [84] which is the result of decreased systolic Ca^2+^ transients, caused by at least three mechanisms that altogether contribute to reduce [Ca^2+^]_mit_ by affecting the activation of the Krebs cycle during increased workload [85]. The first mechanism is associated with a decreased Ca^2+^ load of the SR, with consequent reduction of cytosolic Ca^2+^ transients. The reduction in SR Ca^2+^ load is due both to a lower activity of the SERCA and to leaky RyR2 [84]. During relaxation, Ca^2+^ removal is mainly due to SERCA and the sarcolemmal NCX. Interestingly, in HF, SERCA activity is reduced and, concomitantly, NCX activity increases. As a consequence, more Ca^2+^ is extruded from the cell and less is taken up by SERCA, thus decreasing the SR Ca^2+^ available to be released during subsequent ECC [86, 87]. The second mechanism, associated with contractile dysfunction in failing cardiomyocytes, is the huge increase in cytosolic Na^+^ levels ([Na^+^]_cyt_), which accelerates mitochondrial Ca^2+^ efflux via the mitochondrial Na^+^/Ca^2+^ exchanger (mNCX) [88, 89]. This hampers the activation of Krebs cycle dehydrogenases by Ca^2+^, and it results in pronounced oxidation of NADH to NAD^+^ during transitions of workload [90]. The higher [Na^+^]_cyt_ could be explained by lower Na^+^/K^+^ pump activity, consistent with a decreased Na^+^/K^+^ pump expression in some models of HF [91, 92]. However, this finding was questioned by a more recent study performed in HF rabbit ventricular myocytes demonstrating that the higher [Na^+^]_cyt_ is due to elevated diastolic Na^+^ influx rather than altered Na^+^/K^+^ pump activity [93]. The huge increase in [Na^+^]_cyt_ modifies the direction of the sarcoplasmic NCX transport by inducing the increase in cytosolic Ca^2+^ that partially compensates the decreased SR Ca^2+^ load and release in failing myocytes [89]. On the contrary, no compensatory effects have been detected on mitochondrial Ca^2+^ uptake since the rather slow NCX-mediated Ca^2+^ influx shows less impact on mitochondrial Ca^2+^ uptake [94]. The third mechanism involves changes in the activity of the MCU. Indeed, Michels and colleagues demonstrated that in human cardiac mitochondria from patients affected by HF, the open probability of MCU is decreased [94]. This affects both the energy supply and demand matching and thus the oxidative capacity. This evidence was also observed in isolated cardiomyocytes from a guinea pig model of systolic HF characterized by decreased NADH and NADPH levels and thus in the amount of reducing equivalents necessary for ATP production accompanied by an increase in ROS levels [95].

ROS directly act on cellular structures and activate signalling molecules involved in myocardial remodelling and failure. Indeed, on the one hand low levels of ROS are associated with the activation of the mitogen-activated protein kinase (MAPK) and protein synthesis; on the other hand, high levels of ROS affect ECC in cardiac myocytes [96]. The latter can cause arrhythmias, activate prohypertrophic signalling, and induce apoptotic and/or necrotic cell death through the activation of the mPTP [78]. In addition, mitochondrial ROS play a key role in the development and progression of HF in response to different stimuli such as I/R, pressure overload, and angiotensin II [97]. It has also been demonstrated that oxidative stress stimulates the activity of myocardial metalloproteinases (MMP), a family of proteolytic enzymes that regulates extracellular matrix turnover and that is implicated in the cardiac remodelling after myocardial infarction [98].

The concept that ROS production by cardiac mitochondria is dynamically regulated by Ca^2+^ and ADP and controlled by the redox state of mitochondrial pyridine nucleotides gave rise to the finding that an imbalance between decreased mitochondrial Ca^2+^ uptake and increased cardiac workload triggers oxidative stress [78]. As already discussed above, mitochondrial Ca^2+^ uptake plays a key role in matching ATP production to demand and has a great impact on the redox state of pyridine nucleotides [78]. Therefore, the regulation of mitochondrial Ca^2+^ uptake mediated by the MCU complex in the pathophysiology of heart failure has been extensively investigated in the past years. Indeed, after the characterization of the MCU complex, three different mouse models have been generated: a mouse model with a constitutive global MCU knockout (KO) [99, 100], a conditional cardiac myocyte-specific MCU KO [101, 102], and a transgenic mouse overexpressing a dominant-negative form of MCU (DN-MCU) [103, 104].

Surprisingly, total body knockout of MCU mice is viable and exhibits a very mild phenotype, with slightly smaller body mass than those of wild-type littermates and a slight decrease in skeletal muscle strength and performance [99]. As for the heart, no differences between MCU KO heart and WT littermates were observed [99]. As expected, MCU knockout cardiac mitochondria seem incapable of any rapid mitochondrial calcium uptake and showed alterations in Ca^2+^-dependent oxygen consumption although basal ATP levels were unaltered, suggesting that MCU depletion does not affect basal mitochondrial metabolism [100]. These results can be partially explained by the observation that resting free [Ca^2+^] in knockout mitochondria is only partially reduced [99]. Altogether, these results suggest the existence of alternative mechanisms for Ca^2+^ accumulation in basal conditions, although MCU KO mitochondria were not able to take up Ca^2+^ during cell stimulation [99], fundamental phenomena for responding to variation in energy demands during cell activation. Furthermore, MCU deletion is viable in the outbred CD1 strain, while it results in embryonic lethality in the inbred C57BL/6 strain, suggesting the existence of compensatory mechanisms that allow animal viability only in the mixed background [105]. Another puzzling result is the lack of protection from cell death in the hearts lacking MCU. Indeed, in isolated mitochondria from KO hearts, the addition of large amounts of extramitochondrial calcium did not lead to mPTP activation. Surprisingly following global I/R injury, infarct size was indistinguishable between WT hearts and hearts from MCU KO mice [99], but cyclosporine A, an inhibitor of mPTP, was able to reduce infarct size only in hearts of WT hearts.

As regards the heart-specific MCU KO mice and the transgenic mice overexpressing DN-MCU, a common feature is that, although mitochondria isolated from the heart of these mice are unable to accumulate Ca^2+^, cardiomyocyte function is altered only after *β* adrenergic receptor (*β*-AR) stimulation. This highlights the importance of MCU-mediated mitochondrial Ca^2+^ uptake in sustaining cardiac function during physiological increases in workload. In addition, in DN-MCU mice, it was observed that [Ca^2+^]_cyt_ increases after the positive inotropic and chronotropic responses to *β*-AR stimulation [103, 104]. This finding, corroborated by a study performed on neonatal cardiomyocytes in which MCU was silenced, suggests that MCU may contribute to cytosolic Ca^2+^ buffering in the heart [81, 104].

The genetic ablation of MCU in the heart affects reducing equivalent productions. Since the redox state of NADH is closely linked to NADPH through the activity of a key antioxidant enzyme, the mitochondrial membrane-bound nicotinamide nucleotide transhydrogenase (Nnt), which normally regenerates NADPH from NADH, reduced mitochondrial Ca^2+^ uptake in failing myocytes which increases ROS production [56, 85, 106]. Oxidative stress, in turn, increases [Na^+^]_cyt_ [107] and enhances NCX-mediated Ca^2+^ influx [108], thus generating a vicious cycle of defective ECC, reduced mitochondrial Ca^2+^ uptake, energetic deficit, and oxidative stress, a common scenario observed in HF. Overall, these findings suggest that, in HF, a mismatch of workload and mitochondrial Ca^2+^ uptake causes oxidation of pyridine nucleotides by inducing energy deprivation and oxidative stress [78].

Both the global constitutive MCU KO and DN-MCU mouse models are characterized by a constitutive modulation of MCU activity that may not exclude adaptations to embryonic long-term loss of mitochondrial Ca^2+^ uptake [99, 100, 103, 104]. Phenomena of adaptation have been excluded with the generation of a conditional cardiac MCU KO. Surprisingly, the heart phenotype observed in this mouse was quite similar to constitutive mouse models [101, 102]. However, deletion of MCU in adult cardiomyocytes leads to protection from cell death induced by I/R injury [104]. Furthermore, Ca^2+^/calmodulin-dependent protein kinase II (CaMKII) has been proposed to induce mPTP opening-dependent myocardial death by increasing *I*_MCU_ [109]. CAMKII directly interacts and increases *I*_MCU_. Coherently, transgenic mice with myocardial expression of CaMKIIN, a CaMKII inhibitor, targeted to mitochondria, were protected against I/R injury and showed reduced mitochondrial Ca^2+^ uptake and decreased *I*_MCU_. Nevertheless, these findings have been questioned since the *I*_MCU_ recorded was two orders of magnitude greater than the one previously measured and characterized by high fluctuations incompatible with the low single-channel conductance of MCU [110].

Altogether, these findings demonstrated that the MCU-mediated regulation of mitochondrial Ca^2+^ uptake plays a key role in the onset and progression of HF and thus may be useful for the development of novel treatments targeting mitochondria to ameliorate the progression of the disease.

### 3.2. Skeletal Muscle

Mitochondria play a crucial role in skeletal muscle function by providing ATP largely consumed by actomyosin contraction and SERCA activity. As in the heart, skeletal muscle mitochondria are commonly considered as the predominant source of ROS [111]. Indeed, during exercise, the intense skeletal muscle contractile activity enhances mitochondrial oxidative phosphorylation that increases the oxygen consumption rate and thus ROS production [111]. In detail, it has been demonstrated that O_2_^−^ generation in skeletal muscle increases to about 50- or 100-fold during aerobic contraction [112]. Although ROS have been considered as deleterious species for skeletal muscle tissue, several evidences indicate that they might also play a positive role in physiological processes occurring in muscle cells [111]. Whether a beneficial or detrimental effect prevails depends on several variables among which the most relevant are the duration of ROS flow, the site of ROS production, and the antioxidant status of the cells [111]. In line with this, recently the redox-optimized ROS balance theory has been postulated that assumes that ROS levels depend on the redox state of a cell [113, 114]. In detail, ROS production increases both at high electron fluxes along the ETC, because the ROS production overwhelms the scavenging systems, and when the cellular metabolic state is reduced since reducing equivalents to sustain the antioxidant defences are lacking. Thus, ROS emission from mitochondria is minimal at an intermediate redox state with low ROS production at the ETC, but with sufficient levels of reduced NADPH to neutralize ROS [114]. Although the threshold of physiological ROS concentration above which ROS exert their negative effects has not been yet characterized, different pathways induced or affected by ROS in skeletal muscle has been intensively studied [111]. In particular, low levels of ROS activate signalling molecules such as PGC1-*α*, AMPK, and MAPK that control mechanisms of muscle adaptation such as oxidative metabolism and mitochondrial biogenesis [111]. Importantly, they also exert a self-control mechanism by regulating the activity of antioxidant enzymes. On the contrary, high levels of ROS induce functional oxidative damage of proteins, lipids, nucleic acids, and cell components, a huge increase in intracellular [Ca^2+^] leading to apoptosis and necrosis [111]. Furthermore, several studies demonstrated that dysregulation in ROS production has been considered as causal factors in various muscular pathologies [115–118]. In particular, oxidative stress appears to trigger the myopathic phenotype of malignant hyperthermia susceptibility (MHS) and central core disease (CCD) [119]. MHS is a pharmacogenetic disorder characterized by life-threatening episodes after treatment with depolarizing muscle relaxants while CCD represents one of the most common congenital myopathies [120]. Mutations of the RyR1 gene account for the majority of cases of MHS and CCD [120]. Initial treatment for MHS includes the administration of the RyR antagonist dantrolene, a hydantoin derivate that inhibits the release of Ca^2+^ from SR without stimulating its reuptake [121]. However, since dantrolene suppresses Ca^2+^ release and Ca^2+^ entry, it is not surprising that it could affect protein involved in ECC [122]. Nevertheless, dantrolene is currently the only available pharmacological treatment of MH [121]. Importantly, in several models of these pathologies, it has been observed that the treatment with antioxidant agents ameliorates the muscular phenotype [123]. Indeed, it has been demonstrated that enhanced Ca^2+^ leak from mutant RyR1 increases oxidative/nitrosative stress in the RyR^Y522S^ knock-in mice. This oxidative stress leads to S-nitrosylation of RyR1 which further enhances Ca^2+^ leak from this channel and increases susceptibility to heat-induced sudden death [124].

Dysregulation of [Ca^2+^]_mit_ associated with an increase in ROS production has been proposed as a possible mechanism for skeletal muscle fiber death in Duchenne muscular dystrophy (DMD) [125]. DMD is caused by loss-of-function mutations in the dystrophin gene located in chromosome X [125]. Loss of dystrophin protein increases muscle membrane permeability by inducing a huge increase in [Ca^2+^]_cyt_ that, in turn, induces mitochondrial Ca^2+^ overload [125]. This causes dysfunction of several oxidative phosphorylation enzymes and is accompanied by decreased ATP-synthase activity by influencing both ROS and ATP production in DMD muscles [125]. Increased mitochondrial Ca^2+^ load, occurring in DMD muscles, impairs the ability of mitochondria to reduce free radicals [125] and leads to the onset of apoptotic pathways that culminate in muscle atrophy [125]. This evidence places mitochondria as central participants in the aetiology of DMD, describing the relationship between increased intracellular [Ca^2+^], mitochondrial permeability, and dysfunction culminating in muscle loss [125].

Among the mechanisms that induce the increase in ROS production, the regulation of mitochondrial Ca^2+^ uptake is one of the most relevant. As already mentioned above, the MCU complex plays a key role in regulating Ca^2+^ entry into mitochondria and therefore it might be implicated in the development and progression of different muscular diseases [1]. In particular, the skeletal muscle of total MCU KO mice represents the most affected tissue [99], probably because it shows a much higher *I*_MCU_ compared to the heart (see previous paragraph and [83]). Interestingly, it has also been demonstrated that loss-of-function mutations of the MICU1 gene in humans causes dysfunctional Ca^2+^ uptake and results in clinical and pathological features that usually characterize mitochondrial myopathies, congenital core myopathies, and muscular dystrophies [42]. In particular, muscle biopsies from affected individuals clearly show a myopathic phenotype, characterized by a diffuse variation in fiber size, increased frequency of internal and central nuclei, and clusters of regenerating fibers, without pronounced fibrosis or fat infiltration. Surprisingly, two different MICU1 KO mice display perinatal mortality. One of these models displays an incomplete penetrance, and the KO animals that survive exhibit marked ataxia and muscle weakness, which progressively ameliorate during growth [126]. The physiological relevance of the MCU complex components, and thus the regulation of mitochondrial Ca^2+^ uptake in the onset and progression of muscular diseases, identifies the MCU complex as a potential target for the development of specific pharmacological therapies aimed at both improving the quality of life and increasing the life span of patients.

### 3.3. Neurons

The regulation of mitochondrial shape, volume, number, and distribution within the cells influences mitochondrial function especially in the CNS, where mitochondria show a strategic intracellular distribution, according to local energy demand [127]. Indeed, neurons require extremely precise spatiotemporal control of Ca^2+^-dependent processes, since they regulate vital functions such as transmission of depolarizing signals, synaptic plasticity, and metabolism [1]. For this reason, neurons are extremely sensitive to variations of [Ca^2+^], and even small defects in Ca^2+^ homeostasis, hallmark of aging and neurodegenerative diseases, are able to impair neuronal activity [128, 129]. [Ca^2+^]_cyt_ increases in neurons principally occur through Ca^2+^ entry from the plasma membrane through ligand-gated glutamate receptors, such as the N-methyl-d-aspartate receptor (NMDAR) or various voltage-dependent Ca^2+^ channels (VDCCs), as well as from the release of Ca^2+^ from intracellular stores [130]. The contribution of these sources to intracellular Ca^2+^ in neurons depends on their size, transmitter system, and location in neural circuits (excitatory or inhibitory) [130]. In addition, in presynaptic neurons, Ca^2+^ entry through voltage-operated Ca^2+^ channels promotes the release of neurotransmitters into the synaptic cleft that, in turn, activates receptors located in the postsynaptic plasma membrane by initiating signal transmission [129]. This event generates Ca^2+^ signals that induce specific responses according to the type of receptors that have been activated [129]. Beyond its importance in synaptic transmission, mitochondrial Ca^2+^ uptake guarantees activity-dependent regulation of cellular energy metabolism [131]. Neurons use mitochondrial oxidative phosphorylation to generate ATP, required for cellular metabolism. The major by-product of this process is O_2_^−^ which is dismutated to H_2_O_2_ by the mitochondrial enzyme superoxide dismutase 2 (SOD2) [132]. Since neurons show an extremely high metabolic rate, they produce elevated amounts of ROS in comparison to other organs [132].

In physiological conditions, ROS play active roles in many cellular processes. In particular, in the nervous system, ROS production regulates neuronal development, differentiation, and axon formation [132]. In particular, angiotensin II (Ang-II), brain-derived neurotrophic factor (BDNF), and vascular cell adhesion molecule-1 (VCAM-1) modulate cellular ROS production to regulate neural precursor proliferation and differentiation [133, 134]. Furthermore, it has also been demonstrated that ROS participate in synaptic plasticity as second messengers in several areas of the CNS, including the hippocampus, cerebral cortex, spinal cord, hypothalamus, and amygdala [135–139]. In this regard, it has been shown that repetitive stimuli, by inducing high Ca^2+^ influx, cause an increase in mitochondrial superoxide production. The latter induces the activation of CaMKII and PKA, two kinases involved in synaptic potentiation [132]. Furthermore, it has been demonstrated that increased mitochondrial Ca^2+^ uptake and the consequent stimulation of ROS production plays a key role for the induction of the long-term potentiation (LTP), the principal form of synaptic plasticity in the mammalian brain, thought to endure experience-dependent enhancement of synaptic transmission [132]. In detail, inhibition of MCU blocks potentiation despite the increase in cytosolic Ca^2+^ levels produced after NMDA receptor activation [140]. Mitochondrial ROS, mainly superoxide, activate downstream signalling cascades involving PKA, PKC, and ERK which in turns results in synaptic plasticity of the dorsal horn neurons [140].

Mitochondria-derived ROS levels are regulated by intracellular Ca^2+^ levels. Indeed, ROS increase when mitochondria are exposed to high [Ca^2+^] and [Na^+^], for example, after having sustained NMDA receptor activation [141, 142]. Ca^2+^ influx from N-methyl-d-aspartate (NMDA) receptors triggers mitochondrial activation of caspase 3 which stimulates the synthesis of the myocyte enhancer factor 2 (MEF2) that regulates the transcription of the mitochondrial gene NADH dehydrogenase 6 (ND6), which encodes an essential component of complex I [143]. The MEF2-dependent expression of ND6 reduces cellular levels of the antioxidant enzymes superoxide dismutase and hydrogen peroxidase by increasing oxidative stress [143]. Therefore, dysregulation of mitochondrial Ca^2+^ uptake, and thus a decrease in the rate of ATP production, may influence mitochondrial metabolism and function, thus affecting neuronal activity [131]. In particular, excessive mitochondrial Ca^2+^ accumulation induces an overproduction of ROS that has detrimental effects on neurons [144]. Although mitochondria produce the largest amount of cellular ROS, other sources contribute to the generation of ROS in neurons such as the enzyme neural NOS and the NADPH oxidase. The huge increase in ROS levels induces cellular damage, impairment of the DNA repair system, and mitochondrial dysfunction, all of which are recognized as major determinants of aging and of neurodegenerative disorder development [132].

A recent study highlighted the importance of MCU in controlling excitotoxicity and its implication in NMDA receptor-mediated cell death [145]. In this study, Qiu and coworkers demonstrated that the overexpression of MCU in hippocampal and cortical neurons of newborn mice causes an NMDA-mediated increase in the [Ca^2+^]_mit_ [145]. This increase, in concert with NO production and activation of poly (ADP-ribose) polymerase-1 (PARP-1), leads to the loss of mitochondrial membrane potential which in turn energetically compromises neurons and leads to ROS generation [145]. In addition, knockdown of MCU in neurons causes a decrease in NMDA-mediated mitochondrial Ca^2+^ levels, thus preventing the loss of the mitochondrial membrane potential and excitotoxic cell death [145]. These findings suggest that MCU, and thus mitochondrial Ca^2+^, plays an essential role in neuronal excitotoxicity, although more studies are required to confirm the function of MCU *in vivo*.

### 3.4. Cancer

Tumor formation and progresssion are directly related to mitochondrial dysfunction [146]. Furthermore, reprogramming of mitochondrial metabolism and an aberrant Ca^2+^ homeostasis are considered hallmarks of cancer cells [146]. Multiple lines of evidence highlighted the key role of Ca^2+^ homeostasis deregulation in tumor cell proliferation, apoptosis resistance, tumor development, and metastasis [147]. Although mitochondria exert a key role in cancer progression and Ca^2+^ signalling is altered in a wide variety of tumors, the mechanisms that connect mitochondrial Ca^2+^ homeostasis with malignant tumor formation and growth have not been characterized yet. Recently, Marchi et al. demonstrated that prostate and colon cancers overexpress an MCU-targeting microRNA that, by reducing mitochondrial Ca^2+^ uptake, allows cancer cell resistance to apoptotic stimuli thus increasing tumor cell survival [148]. However, a correlation between MCU overexpression and poor prognosis in breast cancer patients was also recently hypothesized [149]. This study demonstrated that in the MDA-MB-231 cell line, a triple-negative breast cancer model (TNBC), MCU expression correlates with breast tumor size and lymph node infiltration [149]. Coherently, MCU silencing causes a significant decline in [Ca^2+^]_mit_, metastatic cell motility, and matrix invasiveness. Most importantly, in MDA-MB-231 xenografts, deletion of MCU greatly reduces tumor growth and metastasis formation and this is associated with a decrease in mitochondrial ROS production, suggesting that mitochondrial ROS might play a crucial role in cell malignancy regulation by mitochondrial Ca^2+^ uptake [149]. In addition, MCU silencing in TNBC cells downregulates hypoxia-inducible factor 1-alpha (HIF1-*α*) expression, thus negatively affecting the expression of HIF1-*α* target genes involved in cancer progression [149]. Elevated levels of ROS have been detected in almost all cancers including Akt-positive tumors [150]. Akt or more commonly known as protein kinase B (PKB) is a cytosolic protein kinase that regulates cellular energy metabolism and apoptosis through mechanisms that converge on mitochondria or via the phosphorylation of key proteins like the Bcl-2-associated death promoter (BAD) protein. BAD is a proapoptotic member of the Bcl-2 gene family which is involved in initiating apoptosis. Marchi and coworkers demonstrated that Akt phosphorylates MICU1 at the N-terminal domain by affecting MICU1 proteolytic maturation and stability, thus altering mitochondrial Ca^2+^ uptake homeostasis [151]. Akt-mediated phosphorylation of the MCU complex regulator MICU1 may sustain cancer progression by increasing the basal mitochondrial Ca^2+^ level and ROS production [151]. In addition, mitochondrial Ca^2+^ uptake not only represents a fundamental mechanism to regulate cell survival and metabolism but also plays a pivotal role in the regulation of autophagy that plays both a negative and a positive role in cancer [152, 153]. In particular, mitophagy is an essential process that maintains mitochondrial quality and number by the removal of damaged or unnecessary mitochondria using autophagic machinery, thus limiting cellular degeneration [154]. Increasing evidence from different studies supports the concept that dysregulation of mitophagy is an etiologic factor in tumorigenesis [155]. Even though tumorigenesis relies on inhibition of mitophagy, tumor progression likely relies on the presence of functional mitophagy [156–160].

It is important to underline that dysregulation of mitophagy represents a scenario that characterizes not only cancer but also different spectra of diseases including neurodegenerative diseases, motor neuron disorders, autosomal dominant optic atrophy, I/R injury, diabetes, aging, and cancer [161]. The discussion of this aspect is beyond the scope of this review and has been reviewed in greater detail elsewhere.

Altogether, these data reveal the importance of the association between aberrant mitochondrial Ca^2+^ levels and tumor development and strongly suggest that alteration in the activity of the MCU complex components represents a critical checkpoint of metastatic behaviour and thus a potential pharmacological target to combat aggressive cancers.

## 4. Conclusions

Mitochondria are key intracellular organelles that play a fundamental role in energy production and control many cellular processes from signalling to cell death. The function of the mitochondrial electron transport chain, the major source of ATP in the cell, is coupled with the production of ROS that are maintained at physiological levels by highly efficient mitochondrial antioxidant systems. Moreover, these antioxidant defences rely on mitochondrial metabolism that supplies the reducing equivalents needed for their activity.

In the last years, several studies demonstrated that quick changes in ROS levels, coupled with essential cellular functions, are fundamental participants of physiological signalling. Importantly, mitochondrial calcium, by impinging on aerobic metabolism, plays a crucial role in this process, since it joins the cellular activation stimuli and ROS production. This phenomenon plays a crucial role in the maintenance of cellular homeostasis in several tissues, as discussed above.

When the balance between ROS production and clearance is altered by either overproduction of mROS or impairment of the antioxidant defence, mitochondrial dysfunction occurs, leading to the induction of the cell death cascade. Indeed, the overproduction of mROS and the change in mitochondrial redox homeostasis have been shown to be involved in several pathological conditions, which often are associated with mitochondrial Ca^2+^ overload. For these reasons, the physiopathological role of mitochondrial Ca^2+^ uptake and mROS production has been extensively studied in the past years.

The molecular and functional characterization of the MCU complex components highlights the importance of the dynamic regulation of mitochondrial Ca^2+^ in organ physiology. In particular, in this review, we highlighted the contribution of the MCU complex activity, and thus the regulation of mitochondrial Ca^2+^ uptake, in cardiovascular, skeletal muscle, and neurodegenerative diseases and cancer. Although after the discovery of the molecular identity of the MCU complex many studies have confirmed the crucial role of mitochondrial Ca^2+^ signals in the regulation of cell survival, metabolism, and autophagy, many findings are controversial, and many questions are still open. In this regard, the characterization of the mechanisms responsible for the survival of the total MCU KO mouse only in the outbred strain and the analysis of the mechanisms underlying the different phenotypes of the MICU1 KO mice will be fundamental important to finely dissect the physiological role of mitochondrial Ca^2+^. Furthermore, the structural and functional complexity of the MCU complex needs to be clarified. Thus, the study of the physiological role of the different MCU complex components might be useful to better characterize the regulation of mitochondrial Ca^2+^ uptake in different physiopathological conditions thus resulting in the identification of novel therapeutic strategies to cure pathologies characterized by dysregulation of mitochondrial Ca^2+^ homeostasis. In line with this, the discovery of drugs that modulate the activity of the MCU complex shall be extremely relevant for the future development of MCU-targeting therapies.
